# A Case of Incidental Detection of Asymptomatic Bladder Cancer by Transvaginal Ultrasound

**DOI:** 10.7759/cureus.20451

**Published:** 2021-12-15

**Authors:** Shohei Tanabe, Sachiyo Sugino, Kiyoshi Niiya, Kotaro Ichida, Syuji Morishima

**Affiliations:** 1 Obstetrics and Gynecology, Kobe City Medical Center West Hospital, Kobe, JPN

**Keywords:** transurethral resection of bladder tumor, asymptomatic hematuria, transvaginal ultrasonography, papillary tumor, early-stage bladder cancer

## Abstract

We report a case of an incidental diagnosis of early asymptomatic bladder cancer using transvaginal ultrasonography. The patient was a 68-year-old female without urinary symptoms. Transvaginal ultrasonography revealed a 2-cm papillary mass in the bladder mucosa. Urine cytology revealed the presence of atypical cells indicative of malignancy. Cystoscopy revealed a stalked papillary tumor at the apex of the posterior wall of the bladder. Transurethral resection of the bladder tumor was performed, and a 2-cm papillary tumor was resected. A pathological diagnosis of non-muscle-invasive bladder cancer was made.

## Introduction

Bladder cancer is the 10th most common cancer worldwide [1]. It often causes asymptomatic hematuria [2], and cystoscopy and urine cytology are used to detect it. However, these procedures have limitations, especially in diagnosing early-stage tumors [3]. Herein, we report the first case of an incidental diagnosis of early asymptomatic bladder cancer using transvaginal ultrasonography along with a literature review. Consent for the publication of this report was obtained from the patient, and the study was approved by the ethics committee of Kobe City Medical Center West Hospital.

## Case presentation

**Figure 1:**
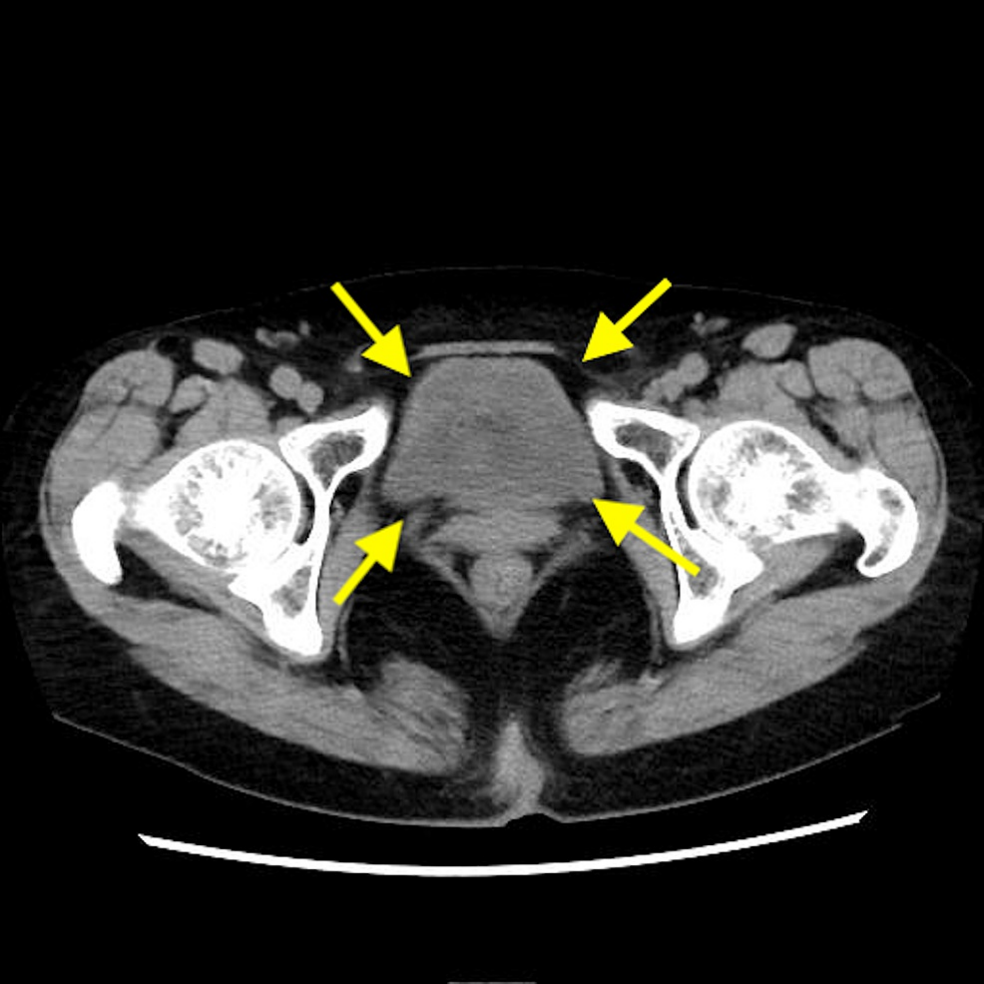
Computed tomography Yellow arrow: bladder There are no findings in the bladder that would raise suspicion of a tumor.

**Figure 2:**
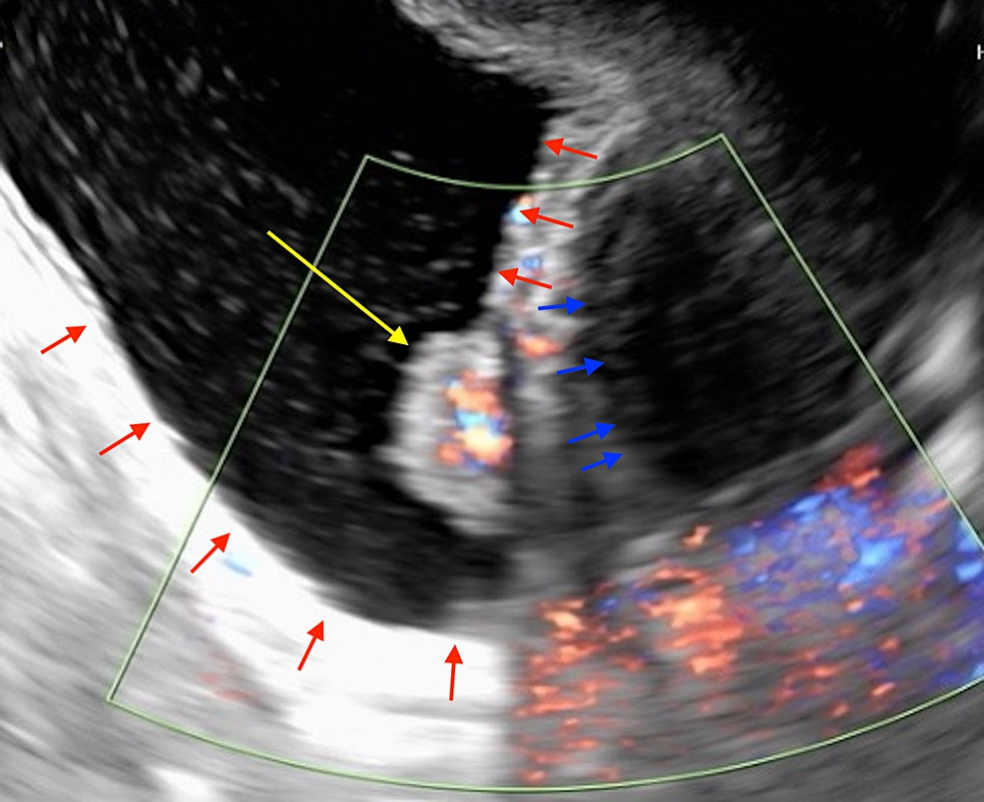
Transvaginal ultrasound image Yellow arrow: bladder tumor; red arrow: bladder; blue arrow: uterus A mass in the bladder mucosa by transvaginal ultrasound was observed.

**Figure 3:**
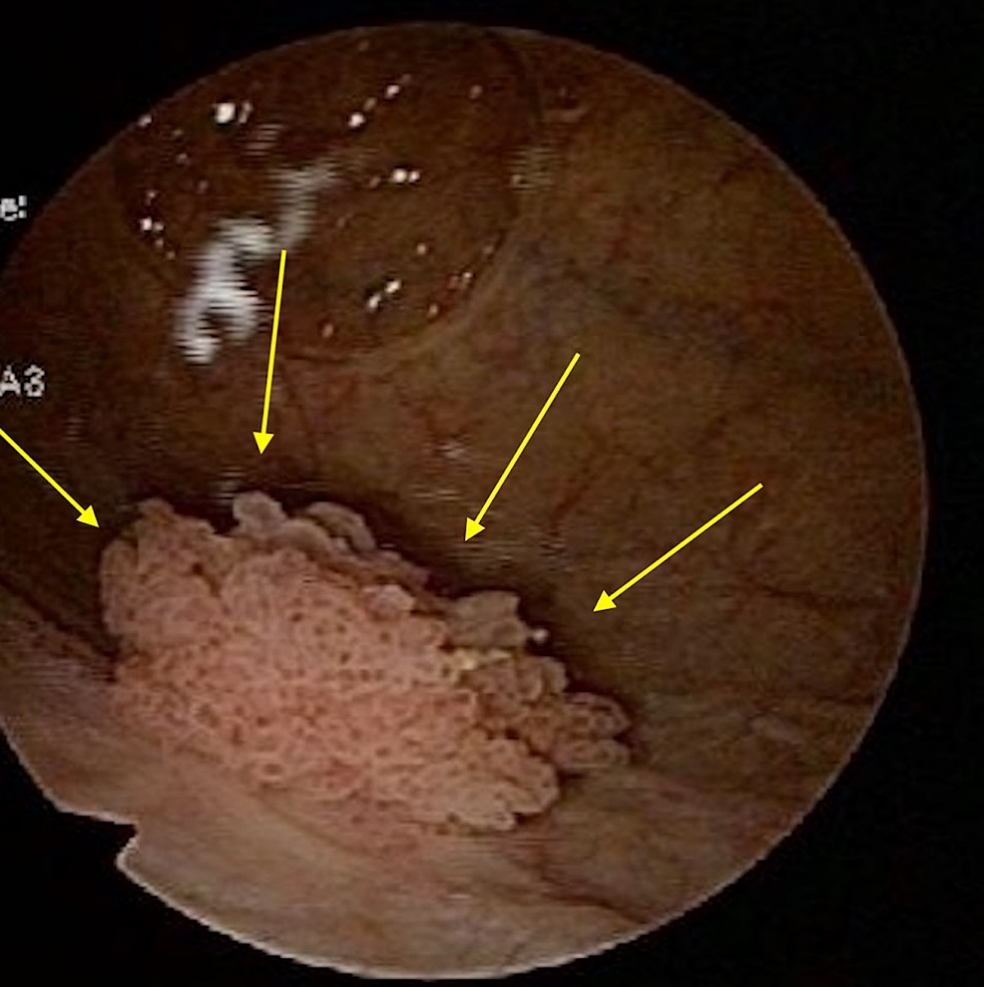
Transurethral resection of the bladder tumor Yellow arrow: papillary bladder tumor

The patient was a 68-year-old female with a G3P2 (G, gravidity; P, parity) pregnancy history who had undergone a pancreatoduodenectomy of the pancreas to remove a tumor (adenocarcinoma) of the duodenal papillae at our hospital five years ago. She underwent computed tomography (CT) during the postoperative follow-up and was suspected of having an ovarian tumor (Figure 1); thus, she visited our Department of Obstetrics and Gynecology. Transvaginal ultrasound showed a mass with abundant internal blood flow in the bladder mucosa (Figure 2). Although the patient had no urinary tract symptoms, an examination by a urologist was deemed necessary. Accordingly, the patient was referred to the Department of Urology, and urinalysis and urine cytology were performed because early-stage bladder cancer was suspected. Urinalysis showed no hematuria, but urine cytology showed dysmorphic cells that were indicative of a tumor. Cystoscopy revealed a stalked papillary tumor at the apex of the posterior wall of the bladder. Transurethral resection of bladder tumor was performed the following month. A 2-cm papillary tumor was found at the apex of the bladder (Figure 3), and the lesion was resected, followed by intravesical chemotherapy administration. The pathological diagnosis revealed that the removed mass was a non-muscle-invasive bladder tumor (transitional cell carcinoma, stage 0a). At the three-month postoperative follow-up, no recurrence was noted. Postoperative CT examination has not yet been performed.

## Discussion

Approximately 90% of patients who develop bladder cancer are aged >55 years, and the average age at diagnosis is 73 years. Men are three to four times more likely to develop the disease than women, and a history of smoking is one of the risk factors for developing the disease [2]. In several cases, bladder cancer has been discovered in older women after visiting an obstetrician and gynecologist due to postmenopausal bleeding [4]. Moreover, bladder cancer is often diagnosed at a more advanced stage in women than in men. One of the reasons for this is the delayed evaluation of hematuria [5].

Bladder cancer can cause gross or microscopic hematuria, and depending on the risk, cystoscopy and imaging of the upper urinary tract can be performed [6]. Screening for bladder cancer in asymptomatic patients without hematuria [2] and asymptomatic women using transvaginal ultrasound is not recommended. However, it has been reported that transvaginal ultrasound can be performed by obstetricians and gynecologists to evaluate cervical cancer for bladder invasion [7] and detect bladder cancer in patients with postmenopausal bleeding [8].

To our best knowledge, this is the first reported case wherein transvaginal ultrasound incidentally indicated bladder cancer in a patient with neither gross nor microscopic hematuria. Since the bladder and ureter, to which particular attention should be paid [9], can be easily evaluated through this method, which obstetricians and gynecologists routinely perform, bladder cancer may be diagnosed early in older women through careful evaluation of the bladder with transvaginal ultrasound. To detect bladder cancer by ultrasound, it is easier to diagnose when the size of the tumor is 0.5 cm or larger. An ultrasound finding suggestive of bladder cancer is an echogenic soft-tissue lesion protruding from the bladder mucosa, which contains internal blood flow on Doppler ultrasonography [10]. However, when observing the bladder, it must be filled with urine to reduce the likelihood of a false positive [11].

## Conclusions

Asymptomatic bladder cancer may be incidentally detected by obstetricians and gynecologists during transvaginal ultrasound. Careful transvaginal ultrasound and observation of the bladder may lead to early diagnosis of bladder cancer in older women.
